# Electrostatic Insect Sweeper for Eliminating Whiteflies Colonizing Host Plants: A Complementary Pest Control Device in An Electric Field Screen-Guarded Greenhouse

**DOI:** 10.3390/insects6020442

**Published:** 2015-05-12

**Authors:** Yoshihiro Takikawa, Yoshinori Matsuda, Koji Kakutani, Teruo Nonomura, Shin-ichi Kusakari, Kiyotsugu Okada, Junji Kimbara, Kazumi Osamura, Hideyoshi Toyoda

**Affiliations:** 1 Plant Center, Institute of Advanced Technology, Kinki University, Wakayama 644-0025, Japan; E-Mail: takikawa@waka.kindai.ac.jp; 2 Laboratory of Phytoprotection Science and Technology, Faculty of Agriculture, Kinki University, Nara 631-8505, Japan; E-Mails: ymatsuda@nara.kindai.ac.jp (Y.M.); nonomura@nara.kindai.ac.jp (T.N.); toyoda@nara.kindai.ac.jp (H.T.); 3 Pharmaceutical Research and Technology Institute, Kinki University, Osaka 577-8502, Japan; 4 Research Institute of Environment, Agriculture and Fisheries, Osaka Prefecture, Osaka 583-0862, Japan; E-Mails: kusakari@mbox.kannousuiken-osaka.or.jp (S.K.); okada@mbox.kannousuiken-osaka.or.jp (K.O.); 5 Research and Development Division, Kagome Co., Ltd., Tochigi 329-2762, Japan; E-Mail: Junji_Kimbara@kagome.co.jp; 6 Technical Development Unit, Panasonic Environmental Systems and Engineering Co., Ltd., Osaka 564-0062, Japan; E-Mail: osamura.kazumi@jp.panasonic.com

**Keywords:** TYLCV, greenhouse pests, physical pest control

## Abstract

Our greenhouse tomatoes have suffered from attacks by viruliferous whiteflies *Bemisia tabaci* (Gennadius) (Hemiptera: Aleyrodidae) over the last 10 years. The fundamental countermeasure was the application of an electric field screen to the greenhouse windows to prevent their entry. However, while the protection was effective, it was incomplete, because of the lack of a guard at the greenhouse entrance area; in fact, the pests entered from the entrance door when workers entered and exited. To address this, we developed a portable electrostatic insect sweeper as a supplementary technique to the screen. In this sweeper, eight insulated conductor wires (ICWs) were arranged at constant intervals along a polyvinylchloride (PVC) pipe and covered with a cylindrical stainless net. The ICWs and metal net were linked to a DC voltage generator (operated by 3-V alkaline batteries) inside the grip and oppositely electrified to generate an electric field between them. Whiteflies on the plants were attracted to the sweeper that was gently slid along the leaves. This apparatus was easy to operate on-site in a greenhouse and enabled capture of the whiteflies detected during the routine care of the tomato plants. Using this apparatus, we caught all whiteflies that invaded the non-guarded entrance door and minimized the appearance and spread of the viral disease in tomato plants in the greenhouse.

## 1. Introduction

Our greenhouse tomatoes have suffered from attack by viruliferous whiteflies, *Bemisia tabaci* (Gennadius) (Hemiptera: Aleyrodidae), over the last 10 years. The greatest economic threat is due to the transmission of damaging plant viruses, especially the Geminiviruses [1,2]. Whitefly is difficult to control with insecticides because they feed and oviposit mainly on the abaxial leaf surfaces [3], and because they have developed resistance to most classes of insecticides used for their control [4,5,6,7]. Physical methods may provide an alternative means of managing the pest, because they would be compatible with other components of integrated pest management, have little impact on the environment, and reduce pesticide use; thus slowing the development of insecticide resistance [8].

The fundamental approach to solve this problem was the application of an electric field screen to the greenhouse windows to prevent their entry [9]. The protection was, on the whole, effective but incomplete because of the lack of a guard at the greenhouse entrance area; in fact, both pests and pathogens entered through the greenhouse door when workers entered and exited [10]. Furthermore, infection by these whiteflies should be reduced to limit the subsequent spread of virus through secondary infestation by the viruliferous whiteflies that multiply on the diseased tomato plants. In our pest control strategy, the window-installed electric field screen is a basic tool for pest exclusion and can be used in combination with supplementary crop protection methods. One such approach was the development and application of an electrostatic insect sweeper. The sweeper is a portable, rod-shape apparatus that enables capture of whiteflies on leaves by means of an electrostatic force generated in the same manner as the electric field screen [11]. In the present study, we describe the construction of the sweeper and its application to the eradication of whiteflies entering through the door of the electric field screen-installed greenhouse for tomato protection.

## 2. Materials and Methods

### 2.1. Insects

Whitefly adults (*Bemisia tabaci*, type B, virus-free) were originally collected from greenhouse-grown tomatoes in Chiba Prefecture, Japan, and maintained at the National Institute of Vegetable and Tea Science in Mie, Japan. The whiteflies were reared on tomato plants in a temperature-controlled greenhouse (26 ± 2 °C, 35%–55% relative humidity) at Kinki University [10]. Male and female adults that multiplied on tomato plants were collected using an insect aspirator (Wildlife Supply, Binghamton, NY, USA). Additionally, three insect species, green peach aphids: *Myzus persicae* (Sulzer) (Hemiptera: Aphididae); Western flower thrips, *Frankliniella occidentalis* (Pergande) (Thysanoptera: Thripidae); and shore flies, *Scatella stagnalis* (Fallen) (Diptera: Ephydridae) were used to test the applicability of the present approach. Adult Western flower thrips and wingless adult female green peach aphids were purchased from Sumika Technoservice (Hyogo, Japan) and reared on water-swollen seeds and 1-week-old broad bean, *Vicia faba* L. “GB-Blend” seedlings, according to the methods of Murai [12] and Murai and Loomans [13], respectively. Hatched winged adult female green peach aphids and adult male and female Western flower thrips were collected with an insect aspirator and used for the following experiments. Adult shore flies were collected from a hydroponic tomato greenhouse and maintained on a lawn of green algae, *Chlamydomonas reinhardtii* (Dangeard) (Chlamydomonadales, Chlorophyceae). This lawn had been cultured on a sponge cube soaked in hydroponic culture solution in a transparent 2-L culture bottle; the bottle opening was covered with a woven net of 0.6 mm mesh [14]. The test insects were held in a temperature-controlled growth chamber (26 ± 2 °C, 35%–45% relative humidity, 16-h photoperiod, with 4000 lux from fluorescent lamps). Average body sizes (length from head to wing tip) of the test insects (20 adults of each species) were 0.79 ± 0.21 mm in the whiteflies, 3.94 ± 0.28 mm in the green peach aphids, 1.59 ± 0.11 mm in the western flower thrips, and 4.21 ± 0.56 mm in the shore flies.

### 2.2. Electrostatic Insect Sweeper

**Figure 1:**
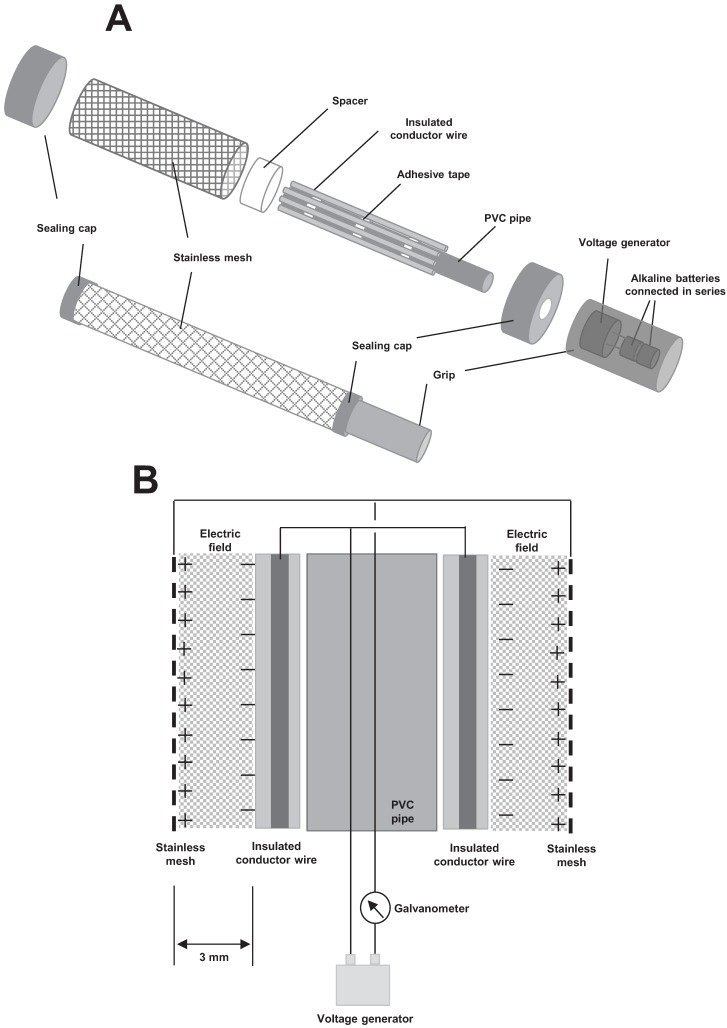
Diagrams of an electrostatic insect sweeper (**A**) and the electrostatic details in the formation of an electric field (**B**).

An iron conductor wire (50 cm length, 2 mm diameter) was passed through a polyvinylchloride (PVC) sleeve (1 mm thickness) to make an insulated conductor wire (ICW). Eight ICWs were held at constant intervals on the outside of a rigid PVC pipe (3 cm diameter) by double-sided adhesive tape wound along the pipe, and then passed through a cylindrical stainless net (2.5 mm mesh size, 40 cm × 10 cm; Figure 1A). The distance between the ICW and net was fixed at 3 mm by placing a spacer (silicone strip with the same thickness) between them (Figure 1B). The ICWs were linked to each other and to the negative terminal, and the net was wired to the positive terminal of a DC voltage generator (MAX-ELECTRONICS Co. Ltd., Tokyo, Japan) that was placed inside a grip. Both ends of the ICWs and the net were sealed with silicone cups. The voltage generator was operated with two 1.5-V alkaline batteries connected in series inside the grip (Figure 1A). The ICW was negatively charged to dielectrically polarize a cover insulator: positively on the iron wire-side surface and negatively on the outer surface of the insulator sleeve [11]. The negative surface charge of the ICW polarized the earthed net to create a positive charge on the ICW side surface as a result of electrostatic induction [15]. An electric field formed between the opposite charges of the ICW and the earthed net (Figure 1B). In the present study, the ICW was negatively charged with voltages of 0.5–10 kV. To measure the mechanical discharge (an occurrence of electric current from the ICW to the net), a galvanometer PC7000 (Sanwa Electric Instrument, Tokyo, Japan) was integrated into a line between the net and voltage generator (Figure 1B).

### 2.3. Insect Capturing Assay

The electrostatic insect sweeper was negatively charged at 0.5–5.6 kV to determine the voltage range that captured all test insects. Adult insects were released onto an abaxial surface of potted tomato plants (1 month old) by an insect aspirator, and then the sweeper was gently slid along the leaves to capture the insects released. Seven to ten adults were used for each species and for each voltage tested. To confirm the successful capture of whiteflies with the ICW, we directed a blower (max. wind speed: 7 m·s^−1^ at the ICW) at the captured insects for 10 min. Experiments were repeated five times, and the data are presented as means ± SD. Significant differences among the data were analyzed using Tukey’s method (see caption to Table 1).

**Table 1:** Percentage of pest insects captured by ICWs of an electrostatic insect sweeper. Seven to ten adults were used for each voltage and for each insect species, and the means and standard deviations of five replicates were calculated. Different letters (a–c) on each vertical column indicate significant differences (*p* < 0.05) according to Tukey’s method.

Pest insects tested	Negative and positive voltages (kV) applied to ICWs									
	0	0.5	0.8	1	1.2	1.5	2	3	5	5.6
Whitefly	0	0	41.6 ± 9.4 ^a^	91.3 ± 6.7 ^a^	100 ^a^	100	100	100	100	100
Western flower thrips	0	0	0 ^b^	73.6 ± 4.9 ^b^	93.5 ± 6.1 ^b^	100	100	100	100	100
Green peach aphid	0	0	0 ^b^	73.3 ± 4.3 ^b^	92.1 ± 7.2 ^b^	100	100	100	100	100
Shore fly	0	0	0 ^b^	32.1 ± 8.7 ^c^	60.4 ± 9.6 ^c^	100	100	100	100	100

### 2.4. Greenhouse Setting

**Figure 2:**
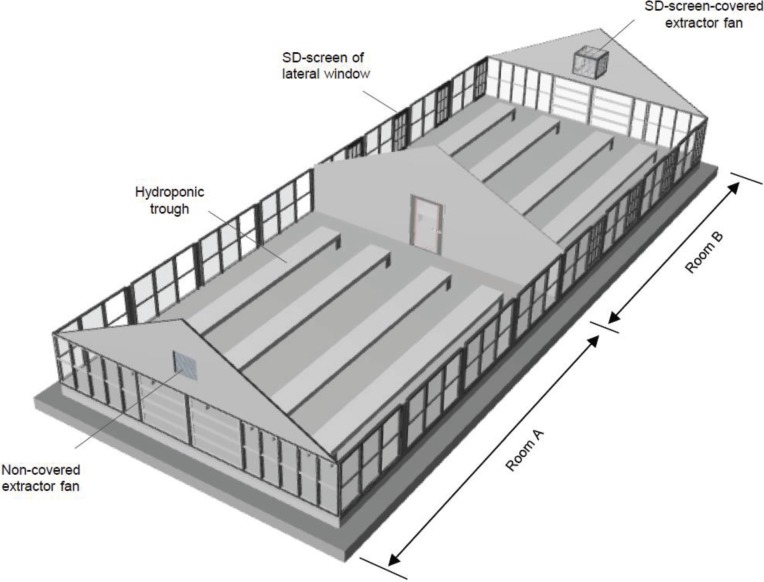
Three-dimensional diagram of the greenhouse, which was divided into two rooms by means of wall partitions. SD screens were attached to the frames of lateral windows on both sides of the right room (room B). The left room (room A) of the divided greenhouse was unguarded, and whiteflies maintained therein propagated on hydroponic tomato plants.

**Figure 3:**
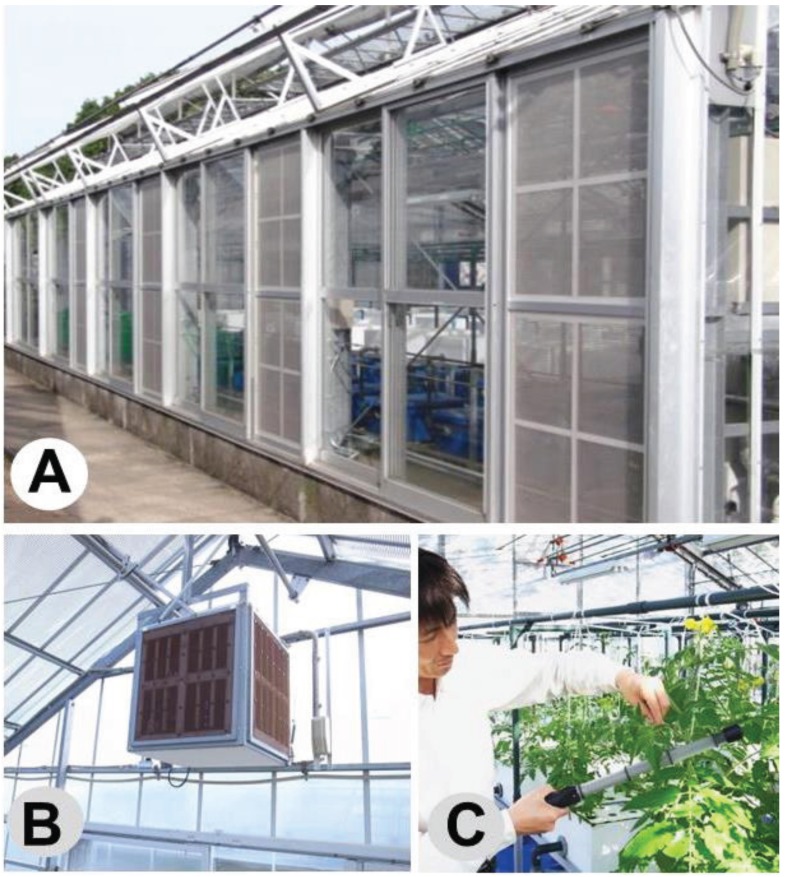
Electrostatic devices used in this study. (**A**) SD screens attached to the lateral-window frames of room B of the divided greenhouse, shown in Figure 2. (**B**) A box of SD screens used to cover an extractor fan in room B. (**C**) An electrostatic insect sweeper operated by a researcher during the routine care of tomato plants.

An A-shaped greenhouse (20 m length, 8 m width, 5 m height at the highest part) was divided into two parts (rooms A and B) by a wall partition with a door (Figure 2). The door of room B was locked throughout the experiment to prevent the entry of whiteflies as researchers entered the greenhouse, whereas the entrance door of room A (the first entrance door) and the inner entrance door of the central partition (the second entrance door of room B) were opened and closed several times per day for ordinary plant care. Single-charged dipolar electric field screens (SD screens) [11] were installed on both side windows of room B. Roof windows were not furnished with the screens because of structural difficulties in doing so, and these remained closed during the experiment. Extractor fans were fitted on the front wall of each half (A and B) of the greenhouse. The extractor fan of room B was covered with a box of SD-screens (Figure 2 and Figure 3B) to prevent the entry of whiteflies through the gap of the fan. An air-circulating fan was attached to a crossbeam in each room, and all fans began operating automatically when the inside temperature reached 30°C. All side windows were automatically closed when the external air speed reached 3 m·s^−1^. The windows were opened again when the wind speed decreased to 1.5 m·s^−1^.

### 2.5. Prevention of Whitefly Entry into the Greenhouse by Window-Installed SD Screens

In total, 400 insect-free, healthy, 40-day-old tomato plants were transplanted to hydroponic culture troughs in room B. On each trough, 10 yellow sticky plates (Y-plates; Arysta Life Science, Tokyo, Japan) were hung from a crossbeam at 1-m intervals to determine the number of whiteflies entering the room. In this experiment, the second and third entrance doors were closed throughout the experimental period to confirm insect repelling by the lateral window-installed SD screens. Three experiments were conducted, one every 2 weeks from July, when vigorous whitefly infestation was confirmed in other tomato greenhouses, to September 2013. At the end of each experiment, we counted the number of whiteflies trapped by the Y-plates.

### 2.6. Greenhouse Assay for Eliminating Whiteflies Entering from an Entrance Door

Tomato plants (30-day-old seedlings) were transplanted to four hydroponic troughs (200 seedlings per trough) in room B, and numerous whiteflies (500–800 adults), collected from neighboring greenhouses, where typical disease symptoms of TYLCV appeared in many cultivated tomato plants, were released on the plants of room A to propagate. A large population of whiteflies in room A was maintained throughout the entire experimental period (6 months) by adding new tomato plants to the hydroponic troughs in that room. In this experiment, a fan blower was placed in room A in front of the second entrance door to produce an airflow towards room B to promote whitefly entry into room B. A fan blower operated at 3 m·s^−1^, while the second door was open. The researchers always went to room B through room A and entered room B 20 times per day for 2 weeks. To trace the whiteflies invading from the entrance door, all leaves of all tomato plants in room B were surveyed carefully every day in the routine care (removal of lateral buds and survey of disease and pest appearance) of the tomatoes. The whiteflies on the leaves were captured with the portable sweeper (Figure 3C) by gently stroking the leaf surface. Three experiments were conducted at 3-week intervals from July to September 2013.

### 2.7. Biotype Determination of Trapped Whiteflies and Virus Detection in Whiteflies

To identify the biotypes of the whiteflies trapped by the Y-plates, we collected single whiteflies from the plates and mixed their homogenates with the reaction mixture from a commercial biotype detection kit (Nippon Gene, Tokyo, Japan) for a loop-mediated isothermal amplification (LAMP) of specific genome sequences of the whiteflies [16]. To detect TYLCV in the trapped whiteflies, we pierced the whiteflies with sterilized toothpicks and then dipped the toothpicks in solution from a commercial TYLCV-detection kit (Nippon Gene) for LAMP of viral DNA [17]. Both experiments were conducted according to the manufacturers’ protocols. In total, 20 whiteflies per Y-plate were collected at random from three plates in each experiment to determine the ratio of biotype-B and -Q whiteflies and to identify carried viruses.

## 3. Results and Discussion

### 3.1. Current Pest and Pathogen Situation in Our Greenhouse Tomatoes

Hydroponic tomato culture is conducted year-round in the greenhouses in Nara Prefecture, Japan, and tomato plants frequently suffer from pathogen infection and/or insect attack. Infection with insect-borne viruses can cause severe damage to tomato plants. Tomato yellow leaf curl virus (TYLCV), carried by the whitefly, represents the most serious threat in high-temperature seasons [9]. Cucumber mosaic virus (CMV), carried by the green peach aphid, and tomato spotted wilt virus (TSWV), carried by western flower thrips, have also occurred occasionally in the past five years. Additionally, shore flies, which inhabit and multiply on alga lawns in sponge cubes soaked with hydroponic culture solution, have been found to transmit rhizosphere pathogens, such as Verticillium wilt; *Verticillium dahliae*; and Fusarium crown and root rot, *Fusarium oxysporum* f. sp. *radicis-lycopersici* [18,19]. Airborne conidia of fungal pathogens pose an additional threat. Powdery mildew (*Oidium neolycopersici*), gray mold (*Botrytis cinerea*), and leaf mold (*Fulvia fulva*) have also occurred commonly on greenhouse tomatoes in the Nara area. Greenhouse tomatoes have been especially affected by year-round powdery mildew infections [20]. The incessant invasion of these pathogen-transmitting insects and fungal conidia necessitates an efficient protection strategy that addresses a wide range of pests and pathogens entering the greenhouse. To overcome this problem, our laboratory has developed an electric field screen technique. [9,11,21,22,23,24,25].

### 3.2. Confirmation of Successful Exclusion of Whiteflies from a Greenhouse by SD Screens

**Figure 4:**
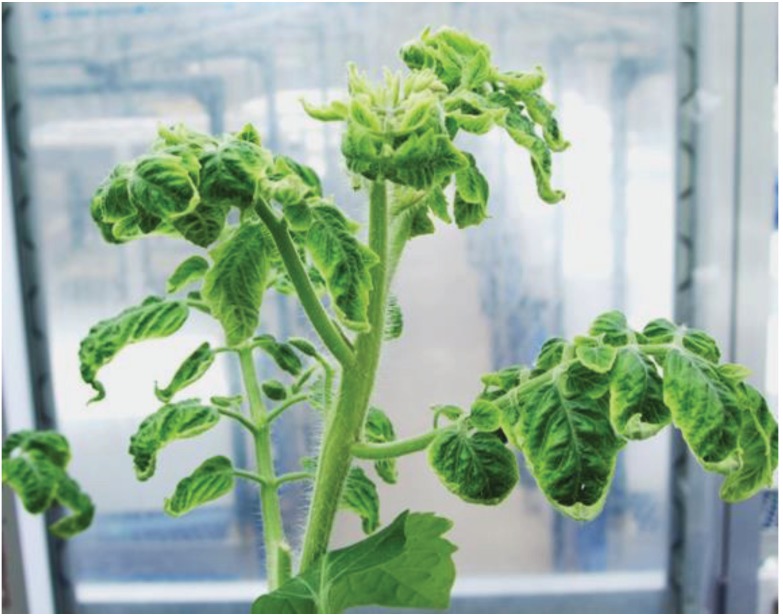
Typical symptoms of TYLCV carried by biotype-Q whiteflies in tomato plants in a hydroponic trough of room A (Figure 2).

One of the main purposes of the current study was to use SD screens to create electrostatically an airy, pest-free space for tomatoes in open-window greenhouses. Previously, we demonstrated that SD screens attached to the window of a greenhouse repelled insects, because the insects avoided the electric field [10,11]; the SD screen, in fact, can capture and repel insects, and the insect-capturing function complements any unsuccessful repelling of insects. Also, in the current experiments, we designed a greenhouse assay to confirm the repelling function of SD screens attached to windows. For this assay, it was useful to monitor the appearance of typical symptoms of TYLCV (yellowing and curling of tomato leaves) in greenhouse tomatoes, because TYLCV-carrying biotype-Q whiteflies are prevalent in our district [26]. In fact, the present PCR-based detection assay showed that the ratio of biotype-Q whiteflies on the Y-plates in room A increased gradually during the three-month experimental period: values of 0.7, 2.7, 7.7, 9.1, 12.1, and 23.4% were detected at two-week intervals. Moreover, the appearance of typical symptoms of TYLCV in the greenhouse tomatoes was another sign of invasion by whiteflies carrying viruses. Symptoms of TYLCV (Figure 4) were detected in 41 tomato plants in room A within two months of transplanting. These results indicated the entry of external virus-carrying whiteflies into room A from the lateral windows, suggesting that room B suffered similarly from invasion by these whiteflies. Nevertheless, room B (in which both entrance doors were locked throughout the experimental period) remained pest-free. Obviously, the SD screens on the windows were successful in terms of preventing entry of external whiteflies to the greenhouse through the windows.

### 3.3. Experimental Setup for Increasing Whitefly Entry through the Greenhouse Entrance Door

A primary aim of this study was ensuring entry of a sufficient number of whiteflies to the greenhouse when workers enter and exit. Our previous paper [10] reported that this type of whitefly entry was frequent, but the number of invading whiteflies was very low (fewer than 10 whiteflies over three weeks). The current situation (*i.e*., heavy infestation of hydroponic tomatoes by whiteflies in one side of a two-room greenhouse) is risky, because numerous whiteflies can enter when the door is opened. In fact, the movement of whiteflies from room A to room B was demonstrated in a control experiment; 85 whiteflies were detected on 15 Y-plates and 28 whiteflies on tomato plants in room B. Most whiteflies entered room B when the wind was blowing in the direction of the second entrance door; the whiteflies were carried inside by the wind when the door opened.

### 3.4. Electrostatic Characteristics of the Sweeper and Its Ability to Capture Insects on Tomato Leaves

The fundamental steps in the present study were to propose an electrostatic apparatus applicable to greenhouse operations and to demonstrate its ability to capture the whiteflies present on tomato leaves. The electrostatic insect sweeper presented was designed for this purpose.

The structure of the sweeper was similar to that of the SD screen [11], although it was modified slightly to improve its portability. In the original SD screen, the ICWs possess earthed metal nets on both sides, and high voltages produced through a Cockcroft circuit [27] of a voltage generator were used to create opposite electrodes; ICWs were negatively charged by the addition of electricity drawn from ground, and the nets were positively charged by pushing electricity (free electrons) of the net to ground [25]. In the current study, we used the voltages to transfer electricity of the net to the ICWs. In this manner, the ICWs and the net became opposite electrodes and an electric field was formed between them (Figure 1B). The most important point of this modification was the removal of the earthed lines from both the voltage generator and metal net, which rendered the apparatus portable.

The flow of the electricity accumulated in ICW depends on the voltage applied to the electrodes, the electrode distance, and the insulation resistance of the ICW cover. An electric current from an insulated conductor depends on the insulation resistance at a given voltage, which determines the level of insulator conductivity [28]. The current was inversely proportional to increases in distance [25]. In this study, we set the distance between the ICWs and the net at 3 mm, according to the original report [11]. The voltage was changed to examine the voltage ranges that would cause a mechanical discharge. Eventually, a constant electric current by the mechanical discharge occurred at >5.7 kV and became larger as the voltage applied increased. From these results, we used voltages between 0.5 and 5.6 kV to determine the range that generated a force strong enough to capture all insects.

The insects in the electric field were attracted to the closer ICW of the sweeper. The mechanism for capturing insects in this field was described previously [25]. In the present study, we examined the ability to capture released adult insects with different body sizes. Table 1 lists the percentage of test insects captured by the sweeper at different voltages (0.5–5.6 kV). Stronger forces of ICWs were necessary to capture the insects with larger body sizes, and the force became stronger with increasing voltage applied to the ICWs. At the same time, there was no significant difference in the capture rate among the three insect species (whiteflies, western flower thrips, and green peach aphids) with a similar body size. At >1.5 kV the force was strong enough that the ICWs captured all adults, regardless of insect size/type. Video data demonstrated the successful attraction of the whiteflies (Supplementary Video 1) to the ICW, and the force at the site was strong enough to capture the insects despite a 7 m·s^−1^ wind speed. These results indicated that a sweeper charged with >1.5 kV could deal with all major pest insects mentioned above under real-world conditions in the greenhouse. At lower voltages, however, the force was insufficient to permanently capture the insects; the captured whiteflies fluttered their legs, twisted their bodies, and then flew away from the ICW (Supplementary Video 2); otherwise, they were blown away from the ICW by a blower. Based on these observations, in the subsequent experiments, the sweeper was charged with 1.5 kV to ensure successful capture.

### 3.5. Practical Application of the Sweeper to Greenhouse Tomatoes 

In our previous paper [20], we presented another electrostatic apparatus (an electrostatic corona discharger) to physically eradicate tomato powdery mildew colonizing tomato leaves. The negatively charged probe of the generator formed a corona discharge at its pointed tip as the probe was brought closer to a leaf surface. Powdery mildew colonies were destroyed by 2-s exposures of the corona discharge. The discharge generator was portable and easy to operate on-site as a part of the routine care of hydroponically cultured tomatoes in greenhouses and provided a non-chemical method to control powdery mildew disease. This successful experience encouraged us to develop a portable pest control apparatus available on-site in a greenhouse. Actually, we expected that the direct trapping method would be effective in collecting the whiteflies entering a greenhouse, because they were almost static on the abaxial leaf surfaces of tomato plants [3].

As mentioned earlier, a large population of whiteflies artificially maintained in room A could be an active source of pests to infest tomato plants grown in the door-connected next room (room B) that was successfully guarded with SD screens. Obviously, the present greenhouse design provided an ideal experimental set-up to create an abundant whitefly invasion from the entrance door to the SD screen-guarded greenhouse. In fact, the whitefly entry was 10-fold higher in number than that of a “natural” invasion of outdoor whiteflies. Under these conditions, we continued to survey all tomato plants in room B and captured whiteflies immediately following their detection on tomato leaves. In total, 82, 96, and 115 whiteflies were trapped during the entire period of the experiment (three weeks) in each of three separate experiments. In all experiments, only one tomato plant showed the typical symptoms of TYLCV; there was no spread of the disease to other tomato plants in room B.

Using the present pest control approach, we failed to achieve complete suppression of viral disease in tomato plants in room B. However, the current method may deserve a more positive evaluation because of its easy operability, low-cost, and environment-friendly application. Additionally, the protective efficacy obtained may be more fully appreciated by considering the following three points: (1) in the present experiment, the greenhouse entrance was always subjected to more frequent invasion of whiteflies than would be the case under normal conditions; (2) the rate of virus-carrying whiteflies was considerably higher in the multiplied population in room A; and (3) the method was effective in suppressing secondary expansion of the disease even when the plants were infected with viruses transmitted by the invading whiteflies. Therefore, we concluded that the present method can be used as a supplementary approach to complement a basic pest exclusion strategy using an electric field screen.

## 4. Conclusions

The electrostatic insect sweeper was developed as a physical method to supplement a pest-exclusion technique for preventing pests entering a greenhouse with an electric field screen installed on its lateral windows. The sweeper possessed a similar structure as the single-charged dipolar electric field screen, and thus captured insects in the same manner. The sweeper was portable and convenient to operate on-site in a greenhouse and was used to eliminate whiteflies from tomato plants, as detected during the routine care of tomato plants in the screen-guarded greenhouse. Whiteflies on the plants were attracted inside a sweeper that was gently slid along the leaves. Using this apparatus, all whiteflies invading from the non-guarded entrance door were captured, which reduced the occurrence and spread of viral diseases of tomato plants in the greenhouse.

## Supplementary Files

Supplementary File 1

Supplementary File 2
